# DEX‐Induced SREBF1 Promotes BMSCs Differentiation into Adipocytes to Attract and Protect Residual T‐Cell Acute Lymphoblastic Leukemia Cells After Chemotherapy

**DOI:** 10.1002/advs.202205854

**Published:** 2023-04-18

**Authors:** Ruinan Jia, Tao Sun, Xin Zhao, Guosheng Li, Yuan Xia, Ying Zhou, Wěi Li, Wei Li, Daoxin Ma, Jingjing Ye, Min Ji, Chunyan Ji

**Affiliations:** ^1^ Department of Hematology Qilu Hospital Cheeloo College of Medicine Shandong University Jinan 250012 P.R. China; ^2^ Shandong Key Laboratory of Immunohematology Qilu Hospital Shandong University Jinan 250012 P.R. China

**Keywords:** adipogenesis, bone marrow mesenchymal stem cells (BMSCs), chemotherapy, minimal residual disease (MRD), T‐cell acute lymphoblastic leukemia (T‐ALL)

## Abstract

T‐cell acute lymphoblastic leukemia (T‐ALL) is an aggressive malignant blood disorder with a high rate of relapse. Patients relapse as a result of minimal residual disease (MRD), which originates from residual T‐ALL cells in the bone marrow microenvironment (BMM). In the present study, it is observed that adipocytes increase dramatically in the BMM of T‐ALL patients after exposure to chemotherapeutic drugs. Then, it is proved that adipocytes attract T‐ALL cells by releasing CXCL13 and support leukemia cell survival by activating the Notch1 signaling pathway via DLL1 and Notch1 binding. Furthermore, it is verified that dexamethasone (DEX) induces adipogenic differentiation by enhancing the expression of SREBF1 in bone marrow mesenchymal stromal cells (BMSCs), and an SREBF1 inhibitor significantly decreases the adipogenic potential of BMSCs and the subsequent ability of adipocytes to support T‐ALL cells in vitro and in vivo. These findings confirm that the differentiation of BMSCs to adipocytes induced by DEX contributes to MRD in T‐ALL and provides an auxiliary clinical treatment to reduce the recurrence rate.

## Graphical abstract

The increasing of adipocytes in the bone marrow of chemotherapy‐treated T‐ALL patients is observed. It is proved that bone marrow mesenchymal stromal cell derived adipocytes attract residual T‐ALL cells by releasing CXCL13 and support their survival via DLL1 and Notch1 binding. Last, it is verified that an SREBF1 inhibitor improves the therapeutic effect of DEX in T‐ALL by reducing adipogenesis.

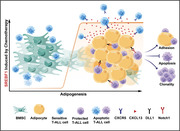

## 1 Introduction

T‐cell acute lymphoblastic leukemia (T‐ALL) is an aggressive malignant blood disorder caused by clonal expansion of variant T cell progenitors, which accounts for ≈25% of ALL cases in adults.^[^ ^1^ ^]^ Due to the use of intensive combination chemotherapy (at least a glucocorticoid, vincristine, and an anthracycline)^[^ ^2^ ^]^ and modulation of treatment tailored to patient response, complete remission rates are high, but relapse often occurs.^[^ ^3^, ^4^ ^]^ Adults younger than 60 years have 5‐year survival rates of only 40–50%, and older patients display an even worse outcome.^[^ ^5^ ^]^ Relapse is believed to develop from undetectable leukemia cells that survive in the bone marrow microenvironment (BMM) after chemotherapy. The BMM is the primary site for minimal residual disease (MRD)^[^ ^6^ ^]^ and supports survival, proliferation, and drug resistance of leukemia cells, presumably contributing to therapeutic resistance and disease relapse.^[^ ^7^, ^8^ ^]^

The BMM comprises cells of multiple lineages, including fibroblasts, osteoblasts, endothelial cells, adipocytes, and mesenchymal stem/progenitor cells. Recent studies have shown that bone marrow adipocytes participate in the development of malignancies by providing energy to solid tumor cells or secreting derived adipokines to protect multiple myeloma cells against chemotherapy‐induced apoptosis and modulating bone metastasis.^[^ ^9^, ^10^, ^11^ ^]^ In addition, the BMM transitions from adipocyte depletion in primary B‐cell acute lymphoblastic leukemia (B‐ALL) to a fully adipocyte‐reconstituted state upon remission.^[^ ^12^ ^]^ Azadniv et al. found that acute myeloid leukemia (AML) had an increased adipogenic potential in BMM compared with normal donor.^[^ ^13^ ^]^ However, no report has shown that adipocytes are increased in T‐ALL patients after chemotherapy and how adipocytes in the BMM influence the survival of T‐ALL cells.

Furthermore, bone mesenchymal stem cells (BMSCs) are precursor cells of adipocytes, and some chemotherapeutic drugs enhance BMSCs differentiation into adipocytes.^[^ ^14^ ^]^ For example, in AML, BMSCs decrease their self‐renewal capability and are prone to differentiate into adipocytes and chondrocytes after treatment with cytarabine (Ara‐C).^[^ ^15^, ^16^ ^]^ Zhang et al. reported that AML cells induced BMSCs toward an adipogenic differentiation propensity.^[^ ^17^ ^]^ It is important to clarify the causes of BMSCs differentiation into adipocytes in T‐ALL patients after chemotherapy and to identify intervention targets for improving MRD in T‐ALL.

In our study, we found an increase in adipocytes in the BMM after exposure to chemotherapeutic drugs by utilizing matched BM biopsies taken from five adult T‐ALL patients at initial diagnosis and after induction therapy and then confirmed that the BMSC‐derived adipocytes attracted T‐ALL cells by CXCL13 and supported T‐ALL cells via the DLL1/Notch1 signaling pathway. Last, SREBF1 was screened out and proven to be a key molecule of dexamethasone (DEX)‐induced adipogenic differentiation. Our findings revealed that the adipogenesis of BMSCs induced by DEX in the T‐ALL BMM contributed to the protection of residual T‐ALL cells, which identified a potential therapeutic target for BMM reconstitution to reduce the recurrence rate.

## 2 Results

### 2.1 DEX Induces BMSC Adipogenic Differentiation in T‐ALL

**Figure 1:**
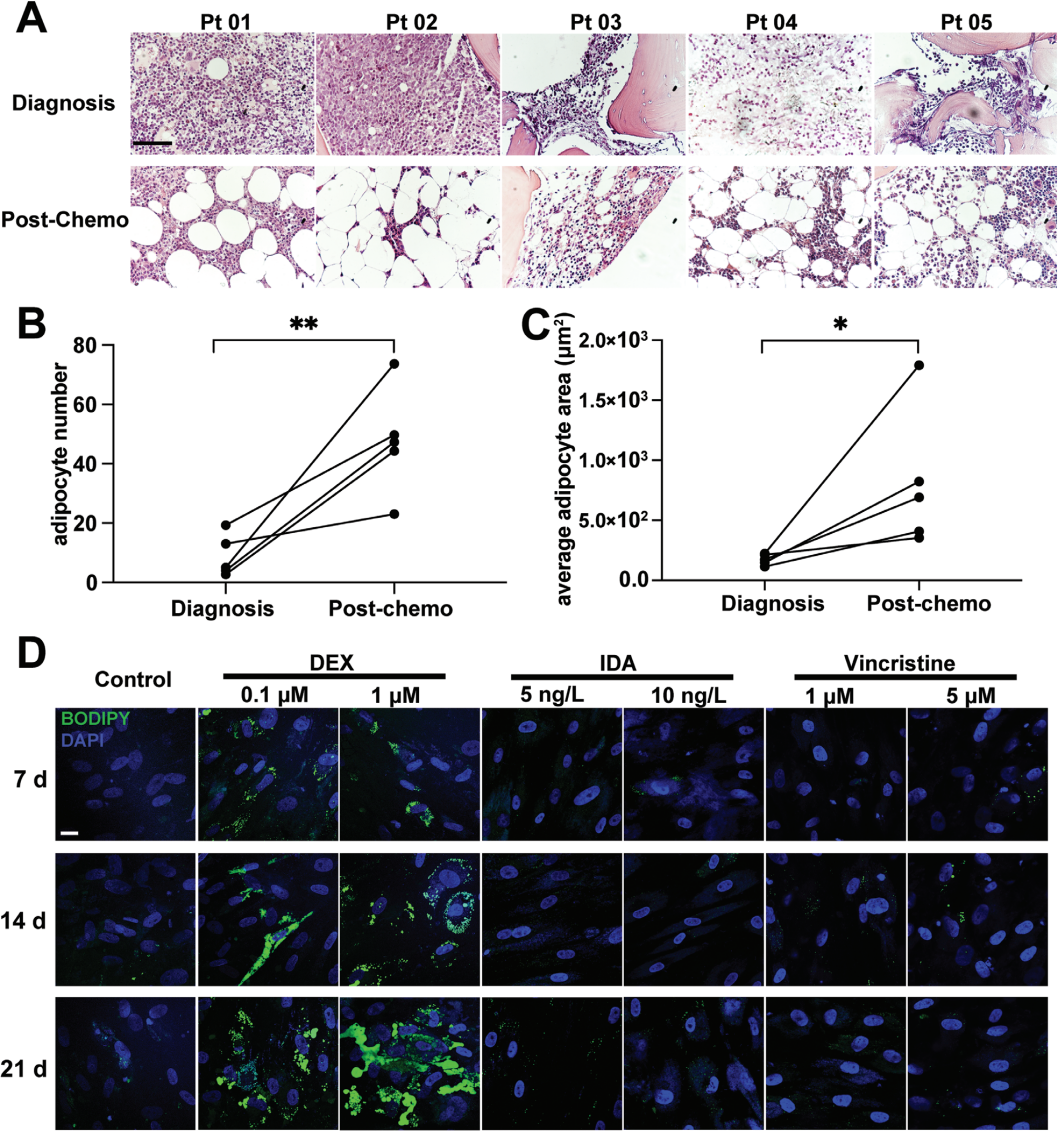
BMSC adipogenic differentiation increases in T‐ALL patients post‐chemotherapy. A) H&E‐stained human BM biopsies from five independent matched pairs of T‐ALL biopsies at diagnosis and post‐chemotherapy (post‐chemo). Bar: 75 µm. B) Adipocyte numbers quantified in diagnosis (*n* = 5) and paired post‐chemo BM biopsies (*n* = 5). Each data point denotes an independent biopsy. C) Adipocyte size quantified in diagnosis (*n* = 5) and paired post‐chemo BM biopsies (*n* = 5). Data points denote the average size for individual adipocytes. D) Lipid‐specific BODIPY staining in BMSCs exposed to DEX (0.1, 1 µm), IDA (5, 10 ng L^−1^), or vincristine (0.5, 1 µm), which are BMSCs from T‐ALL patients. Bar: 25 µm.

To gain insight into the changes in adipocytes in the BMM of T‐ALL patients under chemotherapy, we compared the composition of matched BM biopsies taken from five adult T‐ALL patients at initial diagnosis and after induction therapy. A striking number of adipocytes appeared after T‐ALL therapeutic intervention (**Figure** **1**A), and custom image analysis revealed a profound increase in adipocyte numbers (Figure 1B) as well as major enlargements in adipocyte size (Figure 1C) after induction therapy compared with that at the newly diagnosed stage. Altogether, a large number of adipocytes was generated from the BMM after induction therapy.

Five T‐ALL patients were all treated with the standard chemotherapy regimen, which included vincristine, idarubicin (IDA), and DEX. To demonstrate which component in the three chemotherapy regimens induced the adipocyte differentiation of BMSCs, we performed an evaluation of adipocyte differentiation for BMSCs with these chemotherapy drugs, respectively and continuously, for 7, 14, or 21 days. We first utilized a number of primary BMSCs from T‐ALL patients to increase the objectivity of our results. We set up a control group to observe the baseline differentiation to adipocytes and slight lipid droplet in the control condition was found. The results showed that more BMSCs were differentiated into adipocytes when exposed to DEX, while there was no significant adipogenesis after treatment with vincristine or IDA (Figure 1D). To eliminate the apoptosis in adipogenesis induction process, we examined the viability of BMSCs using a TUNEL assay. No significant apoptosis was observed in BMSCs treated with chemotherapy drugs (Figure S1A, Supporting Information). Then, the adipocytes area was further quantified and the adipocyte conversion rates were normalized to the baseline (Figure S1B, Supporting Information). We observed the chimera which was induced in 1 µm DEX adipogenic medium for 14 days contained half BMSCs and half adipocytes approximately, and it was named “induced BMSC”. The fully differentiated adipocytes which were induced for 21 days contained more than 75% adipocytes and were named “AD” (Figure S1C, Supporting Information). Pre‐treated BMSCs were named “BMSC.” Furthermore, to verify whether adipogenesis was induced by DEX in T‐ALL mouse model, we established NPG mice engrafted model with Jurkat cells and treated them with DEX for 5 weeks, as shown in Figure S1D, Supporting Information. Treatment was initiated when Jurkat cells could be detected in peripheral blood (PB) by fluorescence‐activated cell sorting. At the end of the 5th week, we compared the composition of mouse femurs biopsies taken from control and DEX groups. The results showed a decrease of leukemic burden and an increase of adipocytes in BM treated with DEX (Figure S1E, Supporting Information). Therefore, our results revealed that DEX may be the essential component that directly induces the differentiation from primary BMSCs to adipocytes during the chemotherapy of T‐ALL, which is consistent with the characteristics of BM biopsies from T‐ALL patients at initial diagnosis and after induction therapy.

### 2.2 BMSC‐Derived Adipocytes Attract T‐ALL Cells Via CXCL13

**Figure 2:**
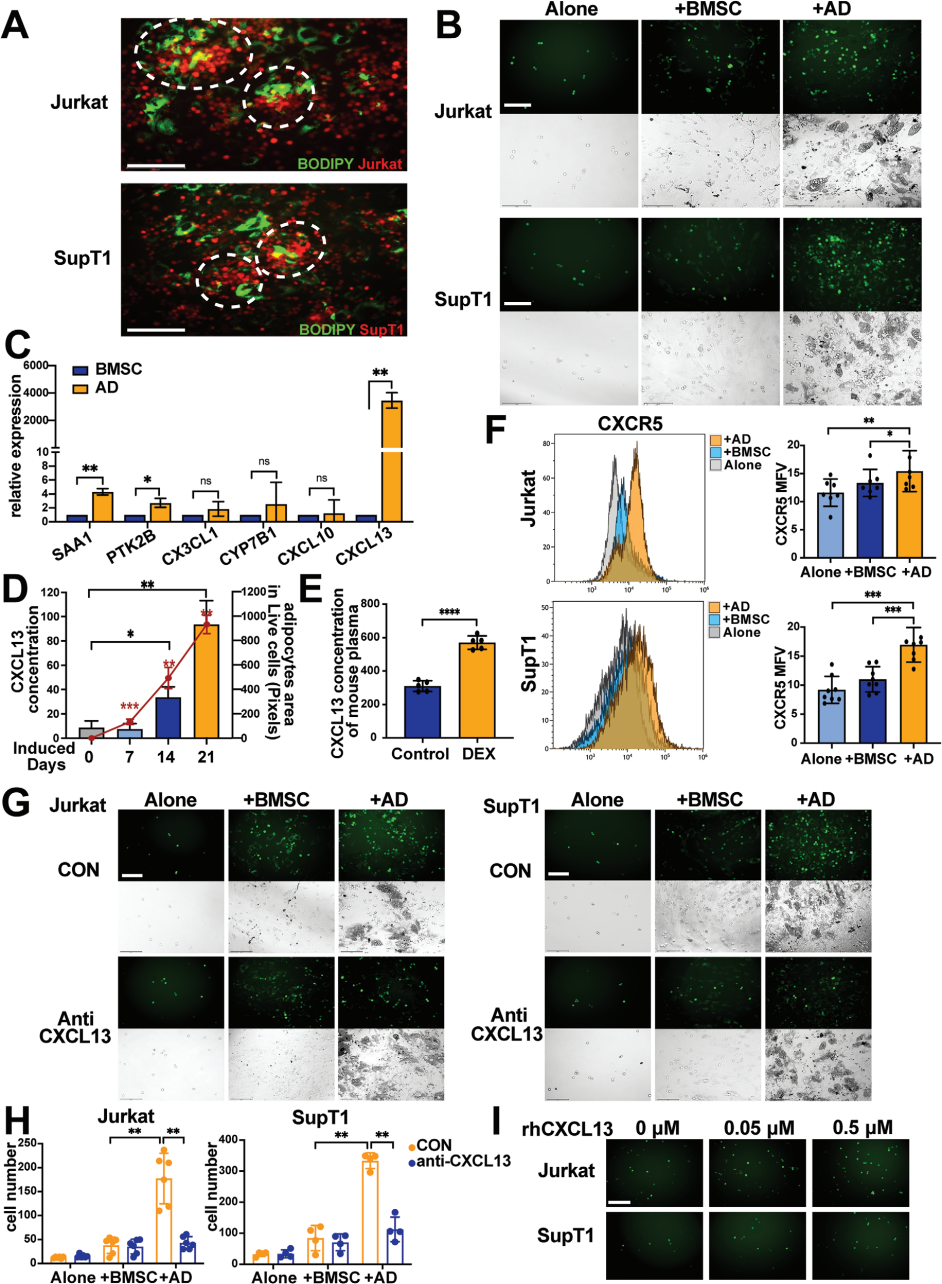
BMSC‐derived adipocytes attract T‐ALL cells via CXCL13. A) Direct coculture systems of T‐ALL cell lines (Jurkat or SupT1 marked by mScarlet fluorescent protein) with induced BMSC. The BODIPY marking adipocytes in green. The circles label the aggregation of T‐ALL cell around adipocytes. Bar: 150 µm. B) The results of the cell migration assay. The upper row shows the GFP‐labeled T‐ALL cells that migrated into the bottom chamber through the Transwell. The lower row shows the coexisting system in the bottom chamber of the Transwell. Bar: 150 µm. C) Relative expression analysis of chemotaxis‐related genes in BMSC and AD from T‐ALL patients by RT‒qPCR. D) The line chart showing the adipocytes area induced for 0, 7, 14, and 21 days. Histogram showing CXCL13 concentrations in the culture medium during adipogenesis using a commercial ELISA kit. E) CXCL13 concentrations of mouse PB plasma in control and DEX groups. F) Flow cytometric analysis of the CXCL13 receptor CXCR5 in T‐ALL cell lines under BMSC or AD coculture relative to monoculture (alone). Each data point denotes a T‐ALL patient BMSC sample. Representative histograms of the mean fluorescence value (MFV) are shown on the left. G) Representative micrographs of the cell migration assay with anti‐CXCL13. The upper row shows the GFP‐labeled T‐ALL cells that migrated into the bottom chamber through the Transwell. The lower row shows the coexisting system in the bottom chamber of the Transwell. Bar: 150 µm. H) The quantified analysis of the cells that migrated into the bottom chamber in (G). Each datapoint denotes a T‐ALL patient BMSC sample. I) GFP‐labeled T‐ALL cells migrated into the bottom chamber in the cell migration assay with rhCXCL13 in the bottom chamber.

To explore the effects of the increased number of BMSC‐derived adipocytes on residual T‐ALL cells, we cocultured T‐ALL cells which were marked by mScarlet fluorescent protein directly with human “induced BMSC,” which contained both BMSCs and adipocytes. The coculture system was in serum‐starved condition to mimic the nutrient‐deprived leukemic BMM. Then, the adipocytes in the coculture system were stained by BODIPY. We found a preferential aggregation of red T‐ALL cells around green adipocytes in a surprise. The results showed that T‐ALL cells preferentially aggregated around adipocytes rather than BMSCs in the coculture system (**Figure** **2**A). On the basis of the results, a cell migration assay was performed via Transwell assays with GFP‐marked T‐ALL cells. Three groups were set up on basal layer: blank, BMSC, AD (induced by 1 µm DEX adipogenic medium for 21days). The number of cells that migrated into the bottom chamber placed by adipocytes exhibited an increase compared with that of the other groups (Figure 2B). All the results proved that BMSC‐derived adipocytes attracted T‐ALL cells.

To identify the key molecules for adipocyte attraction of T‐ALL cells compared with BMSCs, we investigated the differences of chemotaxis‐related molecules between BMSC and AD utilizing qRT–PCR. The results revealed significant changes between BMSC and adipocyte gene expression profiles (Figure 2C). The most significant increase was found in the gene C‐X‐C motif chemokine ligand 13 (CXCL13). To validate the relevance of CXCL13 and the adipogenesis process, we examined the level of CXCL13 in the culture supernatant from BMSC (pre‐treatment), post‐therapy BMSC (induced for 7 days or 14 days with DEX), and AD (induced for 21 days) by ELISA, and the adipogenic differentiation was measured by BODIPY. The results showed that the expression of CXCL13 was increased in 14 and 21 days groups significantly, which was positively correlated with the adipocytes area (Figure 2D). We also detected the CXCL13 level in plasma from T‐ALL mice and found an increase of CXCL13 in DEX group compared with control which was related to an increase of adipocytes in BM biopsies (Figure 2E; Figure S1E, Supporting Information). As CXCR5 is a specific receptor of CXCL13, CXCR5 in T‐ALL cells was measured by flow cytometry after T‐ALL cells were cultured alone or cocultured with BMSC or AD for 24 h. Coculturing with AD enhanced the expression of CXCR5 in T‐ALL cells (Figure 2F). Therefore, CXCL13‐CXCR5 chemokine pairs are important for the attraction of T‐ALL cells to adipocytes.

For further analysis of whether the recruitment of adipocytes was due to the release of CXCL13, the bottom layers were blocked by CXCL13 antibody, and then, the T‐ALL cells were added to the upper chamber. After culture for 24 h, as shown in Figure 2G,H, the number of cells that migrated into the bottom chamber was dramatically reduced. In contrast, when rhCXCL13 was placed in the bottom chamber at different concentrations, the number of cells migrating to the bottom increased with increasing concentrations of rhCXCL13 (Figure 2I). These findings suggest that CXCL13 played an important role in the recruitment of T‐ALL cells.

### 2.3 BMSC‐Derived Adipocytes Support T‐ALL Cells Via the DLL1/Notch1 Signaling Pathway

**Figure 3:**
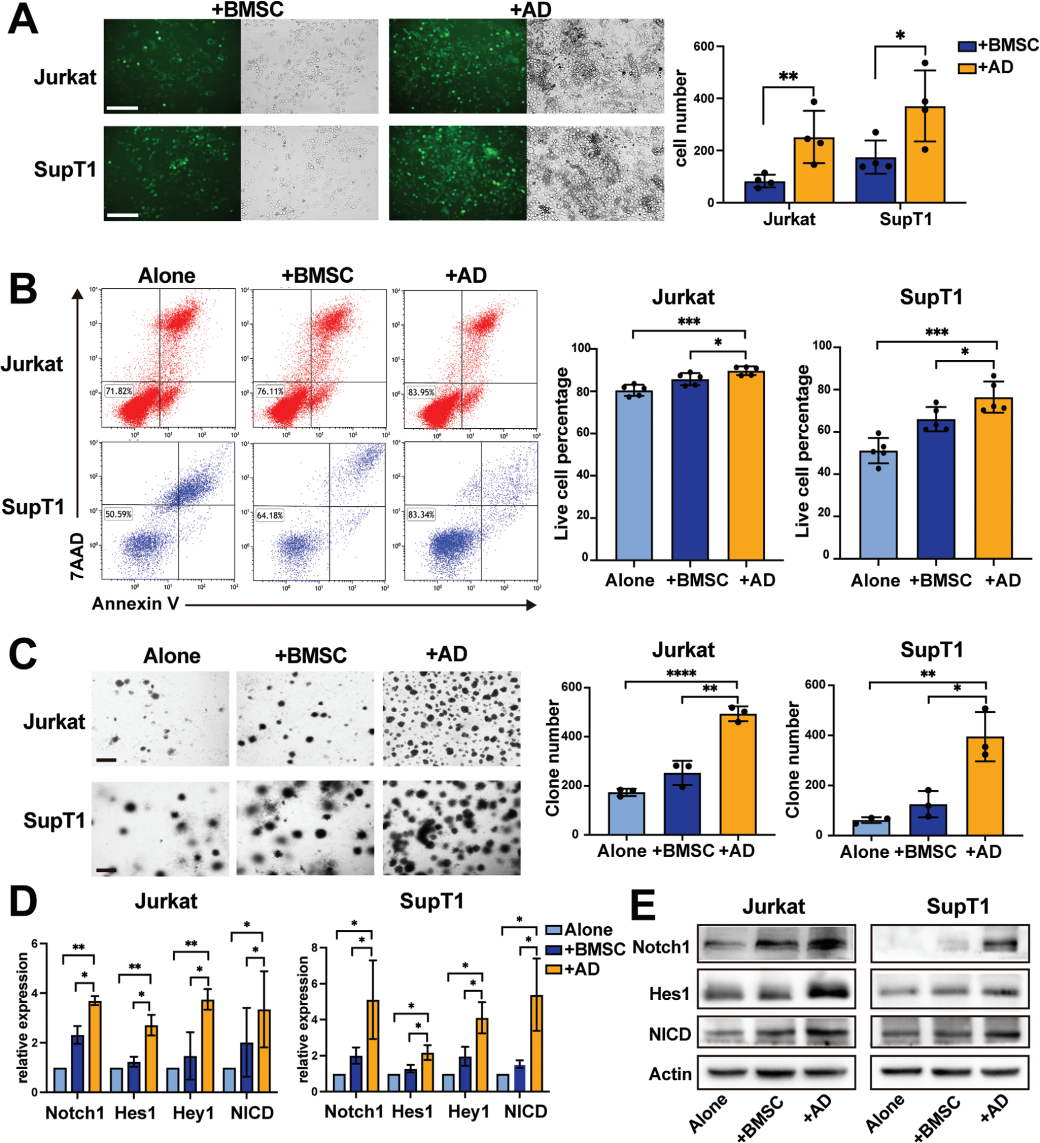
BMSC‐derived adipocytes support T‐ALL cells by activating the Notch1 signaling pathway. A) The number of cells adhering to BMSC or AD. Representative micrographs of GFP‐labeled T‐ALL cells and the coexisting systems are shown on the left. Each data point denotes a T‐ALL patient BMSC sample. Bar: 150 µm. B) Live cell percentage of T‐ALL cell lines from monoculture (alone), BMSC, or AD cocultures in the presence of Ara‐C. Representative cell apoptotic analysis is shown on the left. Each data point denotes a T‐ALL patient BMSC sample. C) The clone numbers of T‐ALL cell lines from monoculture (alone), BMSC, or AD coculture. Representative CFU micrographs are shown on the left. Each data point denotes a T‐ALL patient BMSC sample. Bar: 650 µm. D) Relative expression analysis of Notch1‐related molecules in T‐ALL cell lines from monoculture (alone), BMSC, or AD coculture by RT‒qPCR. Data are presented relative to monoculture (alone). E) Western blot showing the expression level of Notch1‐related molecules in T‐ALL cell lines from monoculture (alone), BMSC, or AD coculture.

To determine whether BMSC‐derived adipocytes support T‐ALL cells, we cocultured T‐ALL cells directly with BMSC or BMSC‐derived AD for 48 h controlled by culturing alone, and cell adhesion, apoptosis with Ara‐C and CFU assays were examined. More T‐ALL cells marked by GFP, as shown in the cell adhesion experiment, were reserved by adipocytes in coculture (**Figure** **3**A). After exposure to Ara‐C, the apoptosis of T‐ALL cells was severely diminished in the coculture system with adipocytes (Figure 3B). Similarly, adipocytes supported higher numbers of CFUs than BMSCs (Figure 3C). Thus, adipocytes could promote T‐ALL cell adhesion, protect T‐ALL cells against the apoptotic effects of Ara‐C, and promote clonal proliferation in long‐term growth. Furthermore, many key components of the Notch1 signaling pathway in T‐ALL cells, such as Notch1, Hes1, Hey1, and NICD, were proven to be upregulated significantly when they were cocultured with AD compared with those cultured alone or with BMSC (Figure 3D,E), suggesting that adipocytes protected T‐ALL by activating the Notch1 signaling pathway.

**Figure 4:**
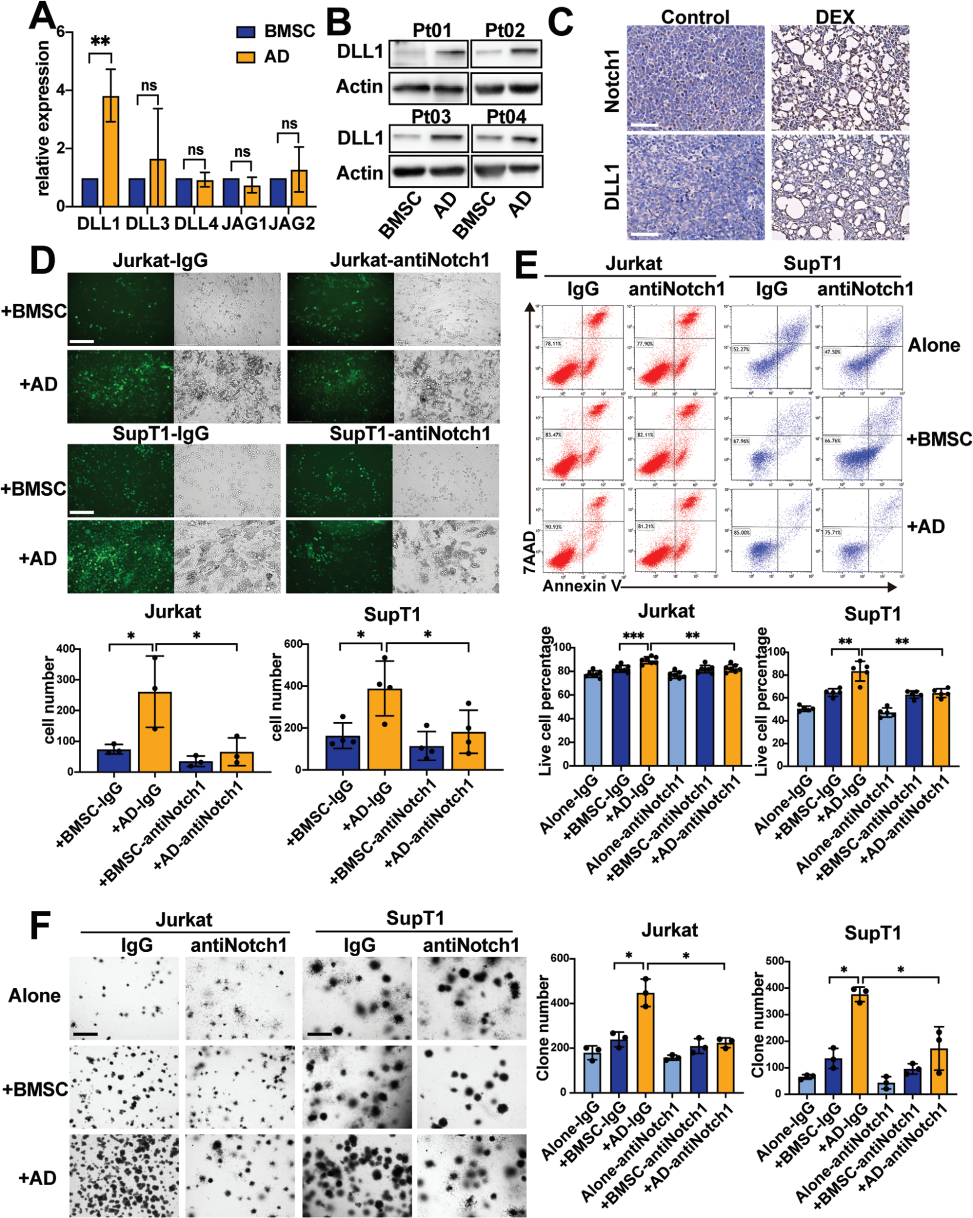
BMSC‐derived adipocytes support T‐ALL cells via the binding of DLL1 and Notch1. A) Relative expression analysis of the ligands of Notch1 (DLL1, DLL3, DLL4, JAG1, and JAG2) in BMSC versus AD by RT‒qPCR. B) Western blot showing the expression level of DLL1 in BMSC versus AD (*n* = 4). C) Histochemistry staining shows the expression level of DLL1 or Notch1 in mouse BM biopsies. Bar: 75 µm. D) The number of cells adhering to BMSC or AD under treatment with a Notch1 blocker. Representative micrographs of GFP‐labeled T‐ALL cells and the coexisting systems are shown on the upper. Each data point denotes a T‐ALL patient BMSC sample. Bar: 150 µm. E) Live cell percentage of T‐ALL cell lines from monoculture (alone), BMSC, or AD cocultures in the presence of Notch1 blocker and Ara‐C. Representative cell apoptotic analysis is shown on the upper panel. Each data point denotes a T‐ALL patient BMSC sample. F) The clone numbers of T‐ALL cell lines from monoculture (alone), BMSC, or AD cocultures under treatment with a Notch1 blocker. Representative CFU micrographs are shown on the left. Each data point denotes a T‐ALL patient BMSC sample. Bar: 650 µm.

Many Notch1 ligands, such as Jagged1 (Jag1), Jagged2 (Jag2), and Delta‐like ligand 1, 3, and 4 (DLL1, DLL3 and DLL4),^[^ ^18^ ^]^ have been reported to be highly expressed in endothelial cells and to activate the Notch1 signaling pathway in target cells. However, no report about the expression of these Notch1 ligands in BMSC‐derived adipocytes was found. Interestingly, only DLL1 was proven to be upregulated in AD (fully differentiated adipocytes) compared with BMSC by qRT–PCR (**Figure** **4**A), which was verified by western blot analysis of 4 T‐ALL patients (Figure 4B). In addition, we examined the expression level of DLL1 and Notch1 in vivo. The results showed that leukemia burden decreased after the treatment of DEX, but the expression levels of DLL1 in adipocytes and the levels of Notch1 in T‐ALL cells around adipocytes were higher (Figure 4C). These results suggested that the Notch1 signaling pathway in T‐ALL cells was activated by BMSC‐derived adipocytes via DLL1/Notch1 binding. Furthermore, we utilized Notch1‐blocking antibody to block the binding of DLL1 and Notch1 in coculture systems. We found that T‐ALL cells treated with Notch1‐blocking antibody lost their ability to attach to adipocytes (Figure 4D) and had a decreased ability to tolerate Ara‐C (Figure 4E). Moreover, the number of CFUs was decreased (Figure 4F). In addition, Notch1 analogue (human recombinant DLL1 protein, rhDLL1) was used in the coculture systems to verify the results. The results showed that the apoptosis induced by Ara‐C in T‐ALL cells alone group was reduced after treatment with rhDLL1, but the apoptosis of T‐ALL cells cocultured with AD was not decreased after rhDLL1 treatment (Figure S2A, Supporting Information). Moreover, after treatment with rhDLL1, the number of CFUs of T‐ALL cells in alone group was also increased, but no difference was found in AD coculture group (Figure S2B, Supporting Information). These observations indicated that adipocytes activated the Notch1 signaling pathway via DLL1/Notch1 binding to enable the survival of T‐ALL cells.

### 2.4 SREBF1 is Important for DEX‐Induced Adipogenic Differentiation

**Figure 5:**
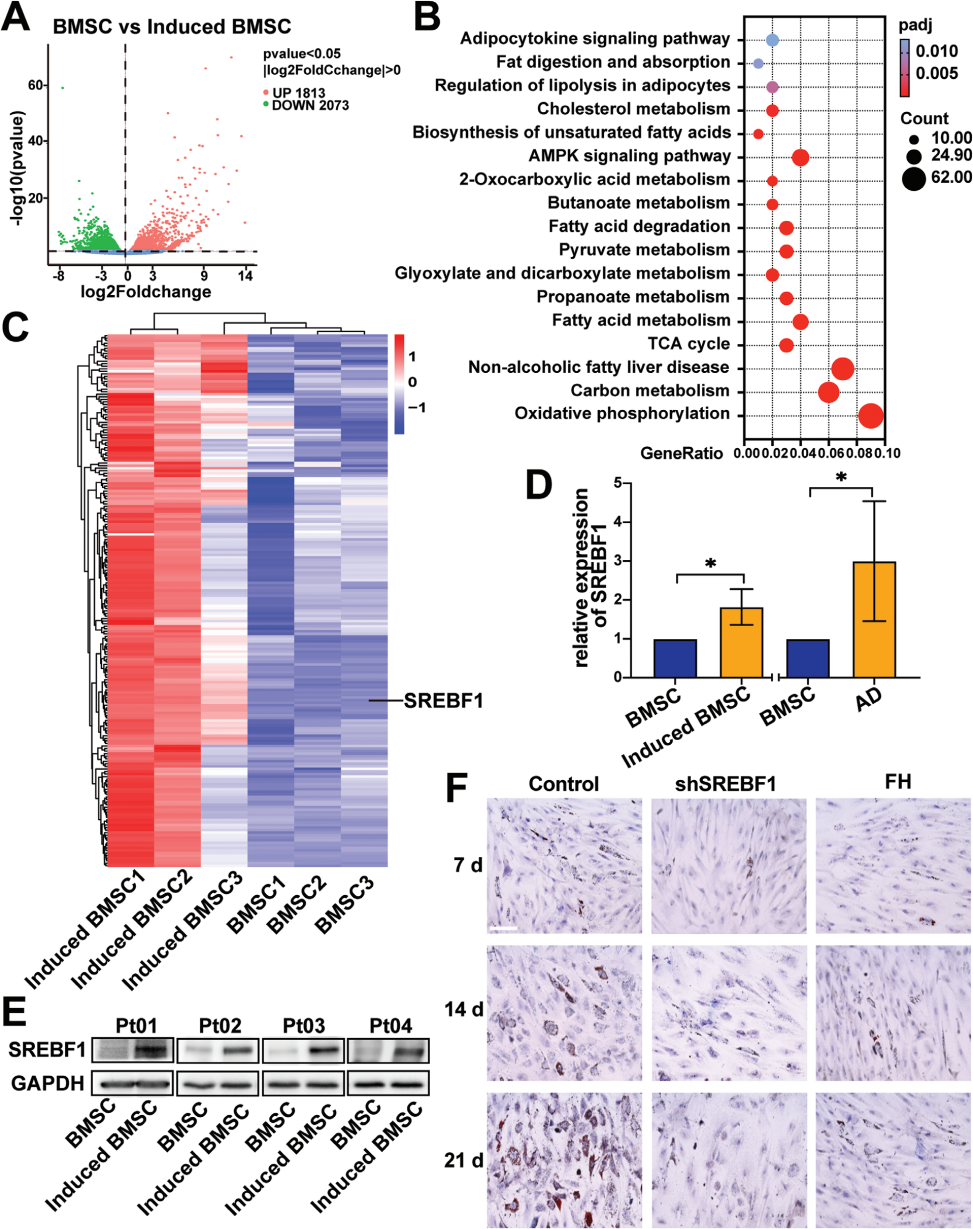
SREBF1 is a key factor for DEX‐induced adipogenic differentiation in T‐ALL. A) Different transcription levels between BMSC and induced BMSC by RNA‐seq. (*n* = 3). B) KEGG enrichment analysis of the upregulated genes in induced BMSC from (A). C) The heatmap of 209 genes related to adipogenesis pathway enrichment. D) Relative expression analysis of SREBF1 in induced BMSC and AD by RT‒qPCR. Data are presented relative to BMSCs. E) Western blot showing the expression level of SREBF1 in BMSC versus induced BMSC (*n* = 4). F) Lipid‐specific Oil red O staining in the process of adipogenesis with shSREBF1 lentivirus transduction or treatment with an SREBF1 inhibitor (Fatostatin HBr, FH) relative to the vehicle control. Bar: 150 µm.

Clarifying the mechanism of adipogenic differentiation induced by DEX is important to avoid the protective effect of BMSC‐derived adipocytes on T‐ALL cells; so, we systematically characterized the potential differences during the process of DEX‐induced BMSCs adipogenesis through unbiased RNA‐seq analysis in three T‐ALL specimens by comparing BMSC and “induced BMSC.” A total of 3886 genes significantly differentially expressed between the two groups were identified (*q* < 0.05, |log2foldchange| > 0, **Figure** **5**A): 1813 upregulated genes and 2073 downregulated genes. Furthermore, Kyoto Encyclopedia of Genes and Genomes (KEGG) analysis of the 1813 genes in induced BMSC was performed. The results showed that these upregulated genes were involved in oxidative phosphorylation, fatty acid metabolism, biosynthesis of unsaturated fatty acids, and so on, which were all associated with adipogenesis (Figure 5B). The genes involved in these enriched pathways were further screened, and the heatmap of the 209 genes is shown in Figure 5C. Interestingly, the expression of SREBF1 (sterol regulatory element binding transcription factor 1, also known as SREBP1) showed a significant upregulation, which has been reported to be important for adipogenic differentiation; thus, SREBF1 might be a target for DEX‐induced adipogenic differentiation. For confirmation of this hypothesis, the expression of SREBF1 was validated by qRT–PCR (Figure 5D) and Western blot analysis (Figure 5E). Then, shSREBF1 lentivirus and an SREBF1 inhibitor (Fatostatin HBr, FH) were used to knock down or inhibit SREBF1 to verify the function of SREBF1 in DEX‐induced adipogenesis by Oil red O staining. As shown in Figure 5F, the number of DEX‐induced adipocytes decreased in the SREBF1 knockdown or inhibited samples. These data indicated a critical role for SREBF1 in the regulation of lipolysis in DEX‐treated adipocytes.

### 2.5 SREBF1 Inhibitor Reduces BMSC Adipogenesis and Subsequent Protection of T‐ALL Cells In Vitro and In Vivo

**Figure 6:**
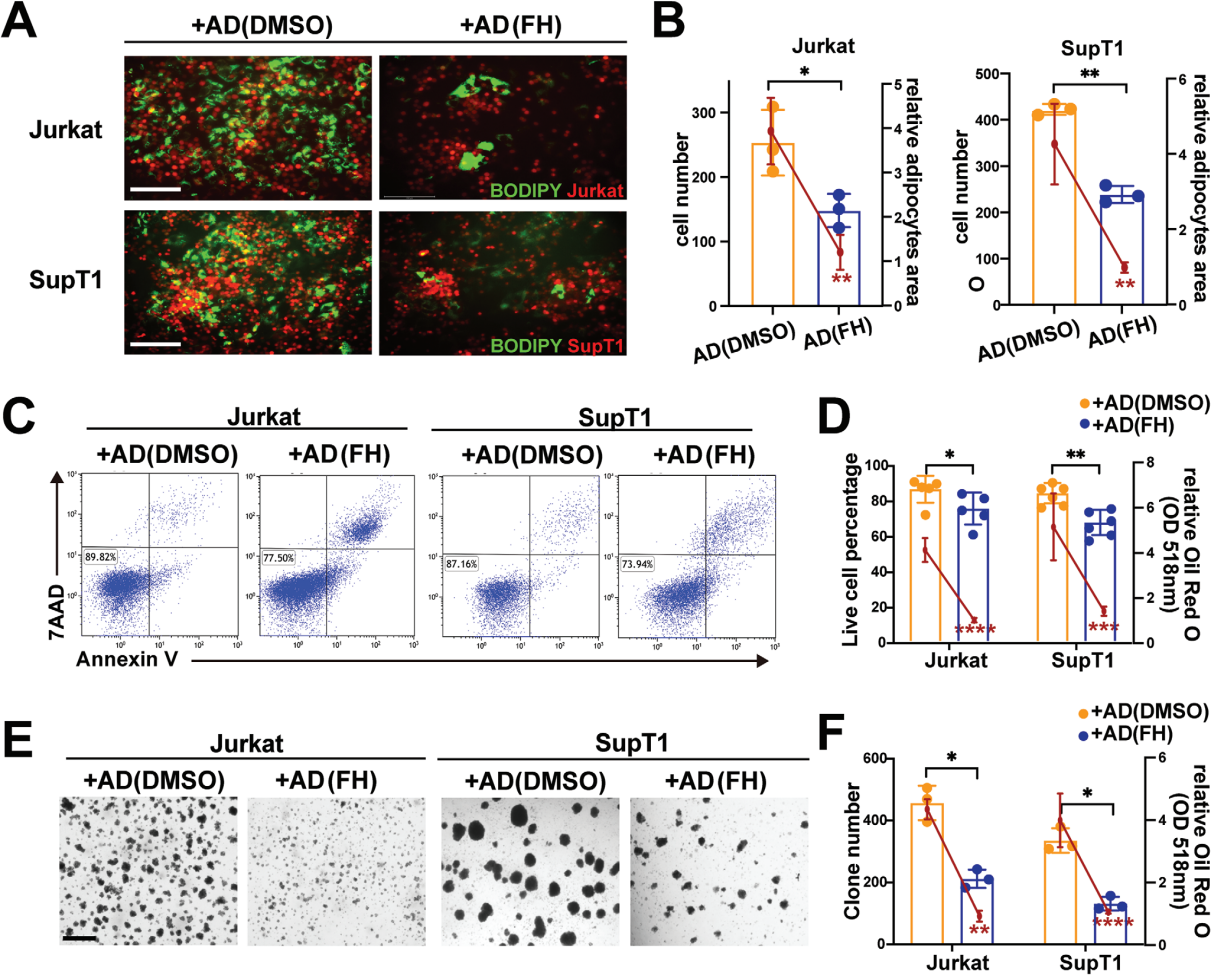
The protection of adipocytes on T‐ALL cells is reduced by inhibiting SREBF1 in vitro. A) Representative micrographs of the coexisting systems in the cell adhesion experiment. T‐ALL cells marked by mScarlet fluorescent protein in red and the BODIPY marking adipocytes in green. The cells adhered to DMSO‐treated AD versus FH‐treated AD. Bar: 150 µm. B) The statistical histograms showing the number of cells adhering to DMSO‐treated AD versus FH‐treated AD. The line chart shows the relative adipocytes area in the coculture systems. Each data point denotes a T‐ALL patient AD sample. C) Representative cell apoptotic analysis of T‐ALL cell lines from DMSO‐treated AD or FH‐treated AD cocultures in the presence of Ara‐C. D) The statistical histograms showing the live cell percentage of T‐ALL cell lines from DMSO‐treated AD versus FH‐treated AD coculture in the presence of Ara‐C. The line chart showing the relative Oil Red O absorbance eluted from the basal cells. Each data point denotes a T‐ALL patient AD sample. E) Representative CFU micrographs of T‐ALL cell lines from DMSO‐treated AD or FH‐treated AD coculture. F) The statistical histograms showing the clone numbers of T‐ALL cell lines from DMSO‐treated AD versus FH‐treated AD coculture. The line chart showing the relative Oil Red O absorbance eluted from the basal cells. Each data point denotes a T‐ALL patient AD sample. Bar: 650 µm.

To investigate the effect of inhibiting SREBF1 in induced BMSC on the support of T‐ALL cells, we induced BMSC treated with or without an SREBF1 inhibitor (Fatostatin HBr, FH) in DEX adipogenic medium for 21 d, and T‐ALL cells were added to the two kinds of BMSC‐derived ADs. Then, cell adhesion, apoptosis with Ara‐C, and CFU assays were performed. In cell adhesion experiment, we quantified adipocytes by BODIPY staining and T‐ALL cells by mScarlet fluorescence protein to study the correlation between adipogenesis and T‐ALL adhesion. The number of adhesive T‐ALL cells after FH treatment was decreased obviously along with inhibited adipogenesis (**Figure** **6**A,B). In apoptosis and CFU‐F assays, to quantify the adipogenesis level, the stromal cells on the basal layer were dyed by Oil Red and detected by absorbance at 518 nm. The number of viable T‐ALL cells exposed to SREBF1‐inhibited AD was decreased along with inhibited adipogenesis (Figure 6C,D). Adipocytes after treatment with FH supported lower numbers of T‐ALL cell CFUs when lipid drops in adipocytes decreased (Figure 6E,F). Thus, the inhibition of SREBF1 reduced the support and protection of adipocytes on T‐ALL by diminishing BMSC adipogenesis.

**Figure 7:**
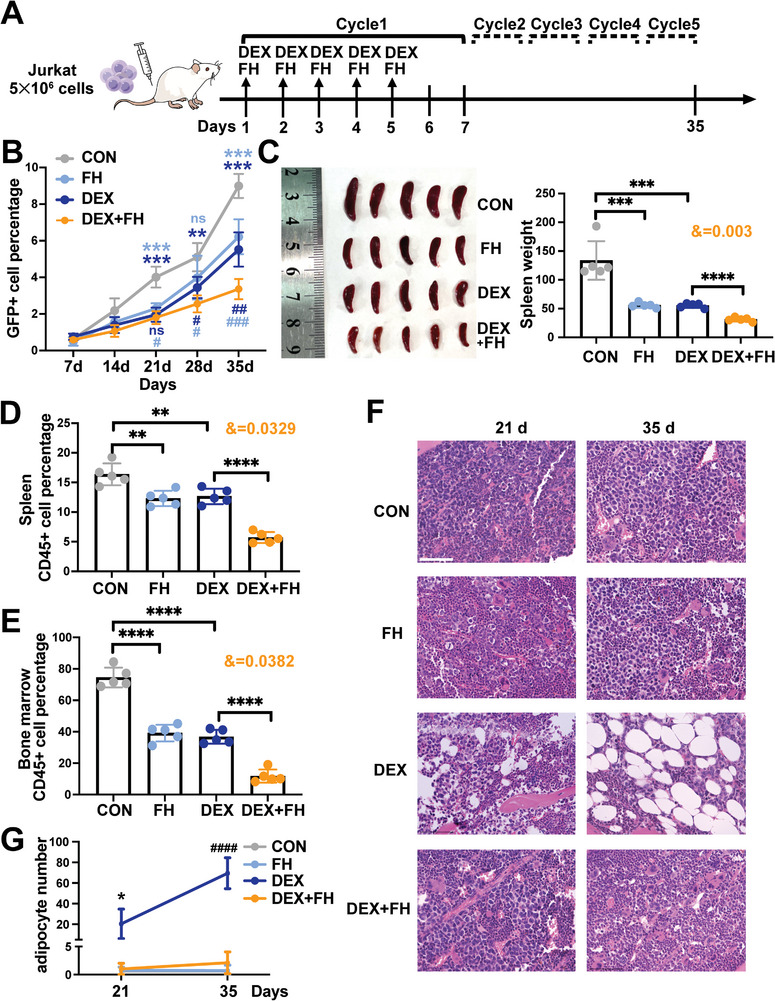
SREBF1 inhibitor reduces BMSC adipogenesis and the subsequent protection of T‐ALL cells in vivo. A) Schematic of the xenotransplantation experiment. B) The GFP+ cell percentage in peripheral blood during the five cycles of treatment using flow cytometry in the control, DEX, Fatostatin HBr (FH), and DEX+FH groups. *, versus the control group; #, versus the combination group. C) Photograph showing the spleen size (left). The statistical histograms show the spleen weight on the right in the four groups. *&*, significant interaction effect; two‐way ANOVA. Each datapoint denotes a mouse. D) Flow cytometric analysis of human CD45+ T‐ALL cell percentage in spleen. Each datapoint denotes a mouse. *&*, significant interaction effect; two‐way ANOVA. E) Flow cytometric analysis of the bone marrow CD45+ cell percentage. Each datapoint denotes a mouse. *&*, significant interaction effect; two‐way ANOVA. F) H&E‐stained mouse BM biopsies from the four groups treated for 21 days or 35 days. Bar: 75 µm. G) The statistical distribution showing the adipocytes numbers of mouse BM biopsies from the four groups treated for 21 days or 35 days. *, versus the combination group for 21 days treatment; #, versus the combination group for 35 days treatment.

To determine whether SREBF1 plays the same roles in vivo as in vitro, NPG mice were engrafted with Jurkat cells and treated with DEX alone, FH alone, or both of them. Treatment was initiated when Jurkat cells could be detected in peripheral blood (PB) by fluorescence‐activated cell sorting. FH and DEX were administered for 5 weeks, as shown in **Figure** **7**A. As presented in Figure 7B, at the end of the 3rd week, the DEX alone and FH alone groups exhibited a meaningful treatment effect, but the DEX + FH group was not significantly different with the DEX alone group. However, at the end of the 4th and 5th week, the effect of DEX + FH combination was highlighted compared with the DEX alone group (Figure 7B). DEX alone or FH alone significantly delayed progression in T‐ALL mice, but the combination effect was significantly greater. In addition, treatment with a combination of DEX and FH significantly reduced the spleen size and weight compared with DEX or FH only (Figure 7C). Similarly, significant reductions in the percentage of human CD45+ cells in spleens and BM were found (Figure 7D,E). The data was statistically analyzed by two‐factor analysis of variance. The results showed that FH or DEX alone group exhibited meaningful treatment effects compared with control. Meanwhile, the combination group had significantly greater interaction effect by two‐factors analysis (spleen weight: *&* = 0.003, spleen burden: *&* = 0.0329, BM burden: *&* = 0.0382), which indicated that FH enhances the therapeutic effect of DEX. More importantly, the results of BM biopsies showed a large number of adipocytes appeared gradually in DEX alone group and a decrease of adipocytes after combination with FH (Figure 7F), which were quantified by counting adipocytes (Figure 7G). Thus, we suggested FH could improve the treatment effect of DEX in vivo by inhibiting adipogenic differentiation of BMSCs.

## 3 Discussion

T‐ALL patients have a high relapse rate of ≈30%, and relapsed cases remain a challenge to treat.^[^ ^2^, ^19^, ^20^ ^]^ MRD is considered an independent prognostic factor in T‐ALL, and MRD‐positive patients have worse clinical outcomes.^[^ ^21^, ^22^, ^23^ ^]^ Our research revealed that adipocytes induced by DEX could attract and protect T‐ALL cells after chemotherapy; so, increased adipocytes contributed substantially to MRD in T‐ALL. Moreover, the protective effect on T‐ALL cells was blocked when we inhibited the function of SREBF1 in the adipogenic process, and inhibition of SREBF1 improved the response to DEX therapy. Thus, our study clarified the protective effect of adipocytes in the BMM on T‐ALL cells and provided a potential strategy to avoid MRD in T‐ALL patients who had been treated with first‐line chemotherapy containing DEX. Our study highlights several important findings.

First, DEX is one of the most effective therapeutic agents in T‐ALL and has been routinely used in hospital settings globally, which is concordant with its established efficacy as a mainstay of multidrug regimens for T‐ALL treatment.^[^ ^24^ ^]^ As a kind of glucocorticoid, DEX; although it has been used for decades in this capacity to treat a variety of diseases, can engender a multitude of tissue‐specific side effects, including growth retardation, osteoporosis, and muscle atrophy, when used at high dosages or for an extended period of time.^[^ ^25^, ^26^ ^]^ In addition, in the BMM, the use of excessive glucocorticoids, such as DEX, promotes the lineage commitment of BMSCs to adipocytes.^[^ ^27^ ^]^ The lineage commitment of BMSCs can be modulated by transcription factors or post‐transcriptional regulators, such as PPAR*γ* and C/EBPs, which initiate and promote adipogenic differentiation of BMSCs. In our study, DEX, an essential component of chemotherapy regimens in T‐ALL, promoted BMSC‐derived adipogenesis in the BMM of T‐ALL both in vitro and in vivo. Inhibition of the mechanism of DEX‐induced adipogenic differentiation is the key to weakening the protective effect of adipocytes on T‐ALL cells and potentiating the long‐term effect of DEX. We discovered a potential key molecule and verified that the inhibitor could potentiate the long‐term effect of DEX, which has strong guiding significance for clinical treatment.

Second, the adipogenic environment is involved in influencing leukemia. For example, in B‐ALL, Heydt et al. found adipocytes impaired the growth capacity and apoptosis of B‐ALL cells and contributed to disease establishment and chemotherapy resistance by targeting post‐transcriptional networks and suppressing protein biosynthesis.^[^ ^12^ ^]^ Cahu et al. focused on thoracic‐ and tail‐derived T‐ALL cells from mouse and found that the effect of stromal cells on leukemia cells could change over time.^[^ ^28^ ^]^ In addition, some findings support the idea that an adipogenic environment is advantageous to AML cell survival. Shafat et al. reported bone marrow adipocytes support the survival and proliferation of AML blasts via a FABP4‐dependent mechanism by transporting fatty acids from adipocytes to leukemia cells.^[^ ^29^ ^]^ In AML, adipocytes likewise enhance light density marrow CFU‐GM and BFU‐E outgrowth.^[^ ^13^ ^]^ Adipocytes have also shown an antiapoptotic effect on AML cells, with an increase in fatty acid oxidation (FAO) along with the upregulation of PPARg, FABP4, CD36, and BCL2 genes.^[^ ^30^ ^]^ In summary, there is no doubt that the interaction between adipocytes and cancer cells is complex and changeable, which is not surprising due to the complexity of leukemia diseases and different experimental backgrounds. However, no previous reports showed that adipocytes affect the spatial chemotaxis of leukemic cells, especially in T‐ALL. We first discovered that adipocytes attracted T‐ALL cells to their surroundings by releasing CXCL13. The Notch1 signaling pathway plays a representative and widespread role in T‐ALL because more than 50% of T‐ALL patients show evidence of mutations in Notch1 or are dependent on Notch1 signaling for their survival;^[^ ^31^ ^]^ so, it is a key point to explore the new and abnormal regulation mechanism. In this research, we found DLL1, as a kind of Notch1 active ligand, was upregulated in DEX‐induced adipocytes compared with BMSCs, and adipocytes promoted the clonality and resistance of T‐ALL cells by activating the Notch1 signaling pathway via DLL1 and Notch1 binding. Our findings have specificity and representativeness in T‐ALL and enriched the perceive of Notch regulation in T‐ALL.

Third, a number of molecules have been reported to be involved in BMSC differentiation, such as FABP4 and SOX9.^[^ ^13^, ^29^ ^]^ On this basis, it is particularly important to clarify the specific molecules regulated by DEX in T‐ALL. SREBF1, known as a transcription factor, binds the sterol‐regulatory element in the enhancer region of several genes that encode enzymes for cholesterol biosynthesis, unsaturated fatty acid biosynthesis, triglyceride biosynthesis, and lipid uptake.^[^ ^32^ ^]^ SREBF1 plays a central role in energy homeostasis by promoting glycolysis, lipogenesis, and adipogenesis via induction of the conversion from acetyl‐CoA to triglycerides.^[^ ^33^ ^]^ The role of SREBF1 in adipogenic differentiation has been known, but we found that DEX could regulate the expression of SREBF1, which was the specific effective target from many adipogenesis regulatory molecules. Moreover, we demonstrated that downregulation or inhibition of SREBF1 diminished adipogenic induction upon exposure to DEX and the subsequent ability of BMSC‐derived adipocytes to support T‐ALL cells. These findings were also observed consistently in murine models. We revealed that SREBF1 played an essential role in BMSC‐derived adipogenesis induced by DEX and could be considered a target to improve the effect of DEX in T‐ALL. Therefore, the utilization of an SREBF1 inhibitor was helpful for therapy in T‐ALL.

## 4 Conclusion

We found that DEX inevitably induced bone marrow adipogenesis while killing malignant cells; as a result, the adipocytes attracted the residual T‐ALL cells by releasing CXCL13 and supported their survival via the DLL1/Notch1 signaling pathway. Moreover, it was proven that an SREBF1 inhibitor could significantly reduce BMSC‐derived adipogenesis and the subsequent contributions to sheltering T‐ALL cells; so inhibiting SREBF1 may be a feasible strategy to solve the dilemma of recurrence in T‐ALL. Our research provides new insight into the survival mechanism of residual T‐ALL cells after chemotherapy from the perspective of bone marrow adipocytes and identifies a potential therapeutic target for aberrant bone marrow adipogenesis to prevent relapse.

## 5 Experimental Section

### Patient Samples and Cell Lines

Bone marrow aspirates and biopsies were collected from patients diagnosed with T‐ALL using procedures approved by the Ethics Committee of Qilu Hospital of Shandong University (DWLL‐2022‐158). Informed consent was obtained in accordance with the Declaration of Helsinki. All the T‐ALL patients met the following inclusion criteria: ≈14–65 years old, percentage of primitive / immature lymphocytes in bone marrow ≥ 20%, diagnosed as T‐ALL by immunophenotyping. The clinical information of the patients is shown in Table S1, Supporting Information. Two human T‐ALL cell lines, Jurkat and SupT1, were cultured in RPMI 1640 medium containing 10% FBS and 1% penicillin–streptomycin at 37 °C with 5% CO2 in a culture incubator.

### Bone Mesenchymal Stromal Cell (BMSC) Culture and Differentiation

Mononuclear cells from bone marrow aspirates were separated by Ficoll‐Paque Plus (Pharmacia LKB Biotechnology) density gradient centrifugation. BMSCs were isolated from T‐ALL bone marrow mononuclear cells by adherence to tissue culture plastic and were then expanded in alpha minimum essential medium (*α*‐MEM) containing 10% FBS and 1% penicillin–streptomycin. Cultured BMSCs isolated at passage 2 or 3 comprised a single phenotypic population.^[^ ^29^ ^]^ Passage 2 BMSCs that reached 90% confluence were allowed to differentiate into adipocytes. To induce adipocyte differentiation of BMSCs, an adipogenic medium of insulin (100 nm), 3‐isobutyl‐1‐methylxanthine (0.5 mm) was prepared with or without one of the chemotherapy drugs (vincristine, idarubicin [IDA], or DEX) in *α*‐MEM containing 10% FBS and 1% penicillin–streptomycin. The differentiated adipocytes in Figures 2, 3, 4, 5, 6 were induced by adipogenic medium containing 1 µm DEX. In this study, “BMSC” were utilized which were pre‐treated BMSCs, “induced BMSCs” which were induced for 14 days with half BMSCs and half adipocytes approximately, fully differentiated adipocytes which were induced for 21 days containing more than 75% adipocytes and named “AD.”

### Coculture Experiments

BMSC and AD were washed twice with serum‐free *α*‐MEM medium for coculture experiments. T‐ALL cells were placed with BMSC or AD at a standard density (1 × 10^6^ cells per mL) in serum‐free medium and were then incubated for 24 or 48 h. T‐ALL cells were separated from the BMSC or AD monolayer by gentle pipetting with PBS, which was repeated twice, and then used in the following experiments. For identification of the T‐ALL cells separated from stromal cells, all the T‐ALL cells in the coculture system expressed GFP or mScarlet fluorescence protein.

### Cell Migration Experiments

A total of 20 000 T‐ALL cells were resuspended in 200 µL of serum‐free medium, with confluent BMSC or AD placed in the bottom chamber of a Corning Transwell system with 8 µm pore polyester membrane inserts, and CXCL13 blocking antibody was added to the bottom chamber. After 24 h, the number of T‐ALL cells in the bottom chamber was observed and quantified.

### Flow Cytometry

PE/Cyanine anti‐human CXCR5 and APC anti‐human CD45 antibodies were purchased from Biolegend (San Diego, USA). Cells were stained for flow cytometry (Beckman Coulter) and then analyzed by Kaluza analysis software.

### Chemokine CXCL13 Testing

The culture medium supernatant was collected from BMSC or AD cultured with serum‐free *α*‐MEM medium for 48 h. CXCL13 measurements were performed using the commercial Human CXCL13 ELISA kit (MULTI Sciences, EK1105‐01). The mouse plasma was collected from peripheral blood and CXCL13 was examined by the commercial Human CXCL13 ELISA kit (Elabscience, E‐EL‐M0190c).

### Apoptosis, Cell Adhesion, and Colony‐Forming Unit (CFU) Assays

T‐ALL cells were sorted and double labeled with an Annexin V//7‐AAD double stain apoptosis detection kit (BestBio, Shanghai, China). Apoptosis was analyzed by flow cytometry according to the manufacturer's instructions. BMSCs were stained with TUNEL (orange fluorescence).

T‐ALL cells were plated on BMSC or AD cell layers at a density of 60 000 cells cm^−2^. T‐ALL cells were allowed to adhere for 24 h at 37 °C in a cell culture incubator. All the media were then collected and the culture systems were washed by gentle pipetting once. The cells that did not adhere to the substrates were sorted, and the adhered cells in the coculture system were observed immediately under a light microscope. The adhered cells were calculated by the average count of five random fields of view.

The T‐ALL cells were harvested and plated in H4230 Enriched MethoCult (Stem Cell Technologies, Vancouver, BC, Canada). After incubation at 37 °C and 5% CO_2_ in a cell culture incubator for 14 days, colony‐forming units (CFUs) were scanned under a light microscope and counted by Image J software.

### RNA Sequencing (RNA‐seq)

Total RNA was extracted from BMSC and induced BMSC of three T‐ALL patients. RNA‐seq was performed using HiSeq 4000. RNA‐seq was performed in one technical run for the three BMSC samples. RNA‐seq reads were aligned to the human genome (hg38). Count files were generated by mapping reads to the human genome (hg38). Differential expression analysis was performed using DESeq2.

### Lentiviral Transduction

Scrambled lentivirus was used as the lentivirus control (control shRNA lentivirus). SREBF1‐targeted shRNA (shSREBF1) and control shRNA lentivirus were purchased from Jikai Biotechnology Co., Ltd. (Shanghai, China). BMSCs were plated at a density of 5 × 10^5^ cells per well in 24‐well plates and infected with lentivirus. After 72 h, BMSCs were then induced to differentiate into adipocytes.

### Oil Red O and BODIPY Staining

Stromal cells were fixed in 4% formaldehyde solution for 30 min, washed with PBS for 5 min, and stained with Oil red O (ORO) solution (Solarbio, China) for 30 min, according to the manufacturer's instructions. Images were captured under a light microscope, and the Oil Red O deposit was eluted with isopropanol and measured by absorbance at 518 nm.^[^ ^34^ ^]^

Stromal cells were fixed in 4% formaldehyde solution for 30 min, incubated with 1 µg mL^−1^ BODIPY for 30 min, and washed with PBS three times. In cell adhesion assays, adipocytes were not fixed and directly stained by BODIPY. Images were captured under confocal microscope and the adipocytes area was quantified by Image Pro Plus software.^[^ ^35^ ^]^

### qRT‒PCR and Western Blot Analysis

Total RNA was extracted from cells using TRIzol reagent (Invitrogen, Carlsbad, CA, United States). Reverse transcription was performed with an M‐MLV RTase cDNA Synthesis Kit (TaKaRa, Japan). Real‐time PCR was performed with the Roche Applied Science LightCycler 480 II Real‐Time PCR System using the SYBR Green gene expression assay (TaKaRa, Japan).

Proteins were separated by 10% sodium dodecyl sulfate‒polyacrylamide gel electrophoresis (SDS‒PAGE) and electrotransferred onto polyvinylidene fluoride membranes. The membranes were blocked in 5% milk at room temperature for 2 h, incubated with primary antibodies overnight at 4 °C, and then incubated with a secondary antibody at room temperature for 1 h. Immunoreactive bands were visualized using an infrared imaging system.

### Xenotransplantation Experiment

Animal studies were conducted in compliance with institutional guidelines and were approved by the Medical Ethics Committee of Qilu Hospital of Shandong University. Male NPG mice (6–8 weeks old; Beijing Aidemo Laboratory, Beijing, China) were intravenously injected with Jurkat cells (5 × 10^6^ cells per mouse). Mice were monitored for evidence of leukemia. Treatment was initiated when the disease could be detected in peripheral blood (PB) by fluorescence‐activated cell sorting. A dose‐finding study was previously performed establishing the following doses: 1 mg kg^−1^ (DEX) and/or 15 mg kg^−1^ (Fatostatin HBr, FH).^[^ ^36^, ^37^, ^38^ ^]^ FH and/or DEX were administered i.p. five days per week (Figure 7A). The Jurkat burden was detected in the PB each week. At the end of 5‐week treatment, mice in each treatment group were sacrificed, femurs and spleens were isolated, and the burden of leukemic cells was analyzed. During the treatment process, the proportion of adipocytes in BM of femurs was evaluated at the end of the 3rd week and 5th treatments.

### Histology and Histochemistry

The femurs were isolated and fixed in 4% formaldehyde for paraffin embedding and sections. Hematoxylin and eosin (H&E) staining was performed as per standard protocols. The adipocytes were white and nearly round, without erythrocytes inside and with a smooth membrane boundary with the surrounding tissue. Anti‐DLL1 and anti‐Notch1 antibodies were used as primary antibodies for histochemistry assays.

### Statistical Analysis

Sample size was chosen *n* ≥ 3. All statistical tests were performed with GraphPad Primer 9. The data are presented as the mean ± standard deviation. Student's *t* test was used for comparisons between two groups. Two‐way ANOVA was used to compare the inter‐differences of two factors. *P* value of less than 0.05 was considered statistically significant.

## Conflict of Interest

The authors declare no conflict of interest.

## Author Contributions

R.J. and T.S. contributed equally to this study. T.S. and M.J. designed the research; R.J., Y.X., and Y.Z. performed the experiments; R.J., X.Z., and Wěi.L. analyzed data; R.J., T.S., and M.J. wrote the manuscript; R.J., Wei.L., J.Y., and M.J. collected clinical samples. G.L. and D.M. provided scientific and technical support. T.S., J.Y., M.J., and C.J. supervised and coordinated all aspects of the research. All authors revised, read, and approved the final manuscript.

## Supporting information

Supporting Information

## Data Availability

The data that support the findings of this study are available from the corresponding author upon reasonable request.
