# Supplementary material for: DEX‐Induced SREBF1 Promotes BMSCs Differentiation into Adipocytes to Attract and Protect Residual T‐Cell Acute Lymphoblastic Leukemia Cells After Chemotherapy
Source: Adv Sci (Weinh). 2023 Apr 18;10(19):2205854. doi: 10.1002/advs.202205854 (PMC10323667; doi:10.1002/advs.202205854)
Supplement: Supplementary file 1 — Supporting Information [file ADVS-10-2205854-s001.pdf]

ADVANCED SCIENCE
Open Access

Supporting Information

for *Adv. Sci.*, DOI 10.1002/advs.202205854

DEX-Induced SREBF1 Promotes BMSCs Differentiation into Adipocytes to Attract and Protect Residual T-Cell Acute Lymphoblastic Leukemia Cells After Chemotherapy

Ruinan Jia, Tao Sun, Xin Zhao, Guosheng Li, Yuan Xia, Ying Zhou, Wěi Li, Wei Li, Daoxin Ma, Jingjing Ye, Min Ji* and Chunyan Ji**

Supplementary Figures and Table

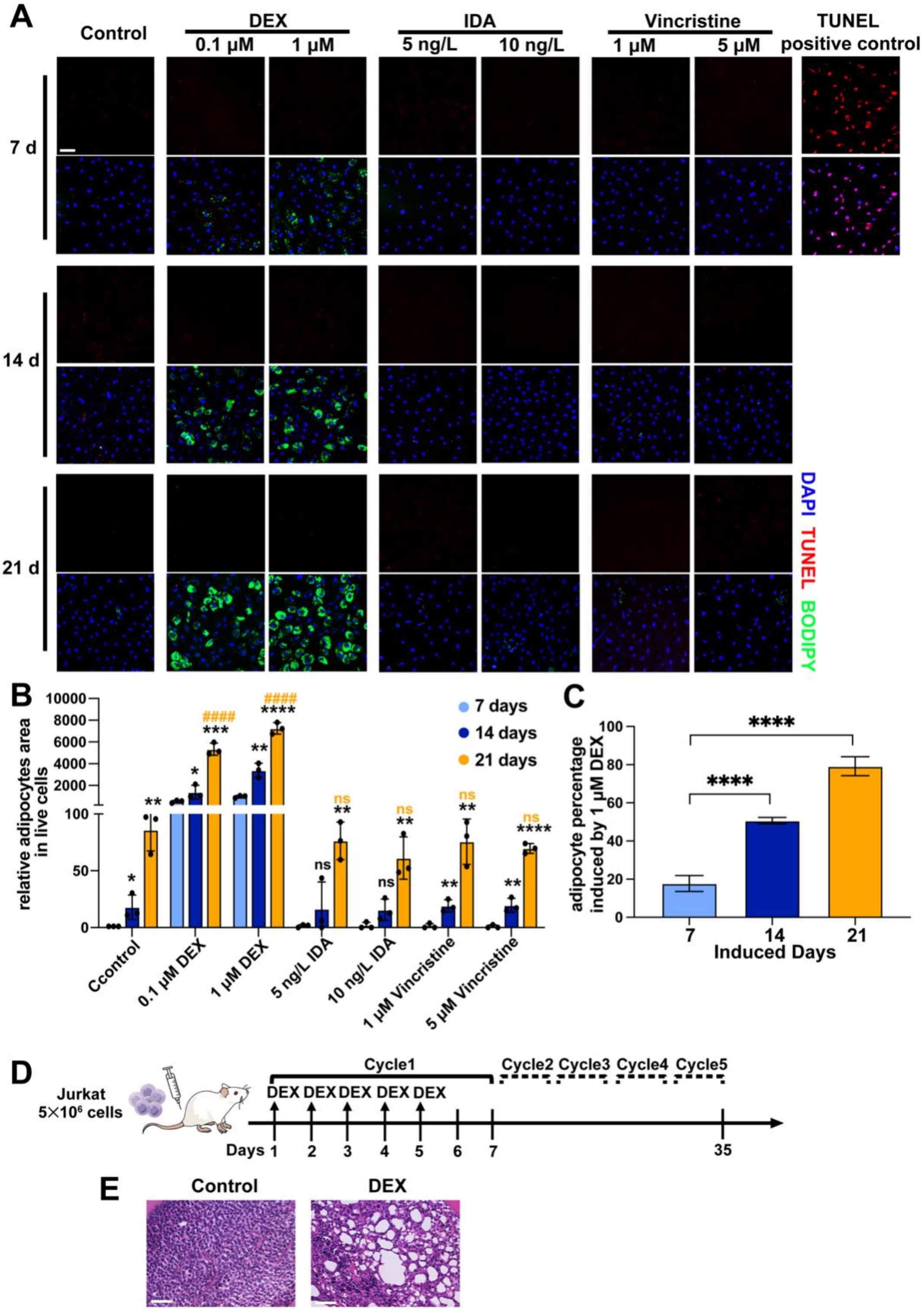

Supplementary Figure 1. BMSC adipogenic differentiation increases in T-ALL patients post-chemotherapy

(A) Lipid-specific BODIPY staining and apoptosis TUNEL staining in the process of BMSC adipogenesis exposed to DEX (0.1 μM, 1 μM), IDA (5 ng/L, 10 ng/L) or vincristine (0.5 μM, 1 μM), which the BMSCs are from T-ALL patients. Bar, 50 μm. (B) The quantified analysis of the relative adipocytes area in live cells induced for 7, 14 or 21 days in A. *, 14 or 21 days vs. 7 days; #, vs. The control group induced for 21 days. (C) The quantified analysis of the adipocytes percentage induced by 1 μM DEX adipogenic medium for 7, 14 or 21 days. (D) Schematic of the xenotransplantation experiment. (E) H&E-stained mouse BM biopsies from the control and DEX groups treated for 35 days. Bar, 75 μm.

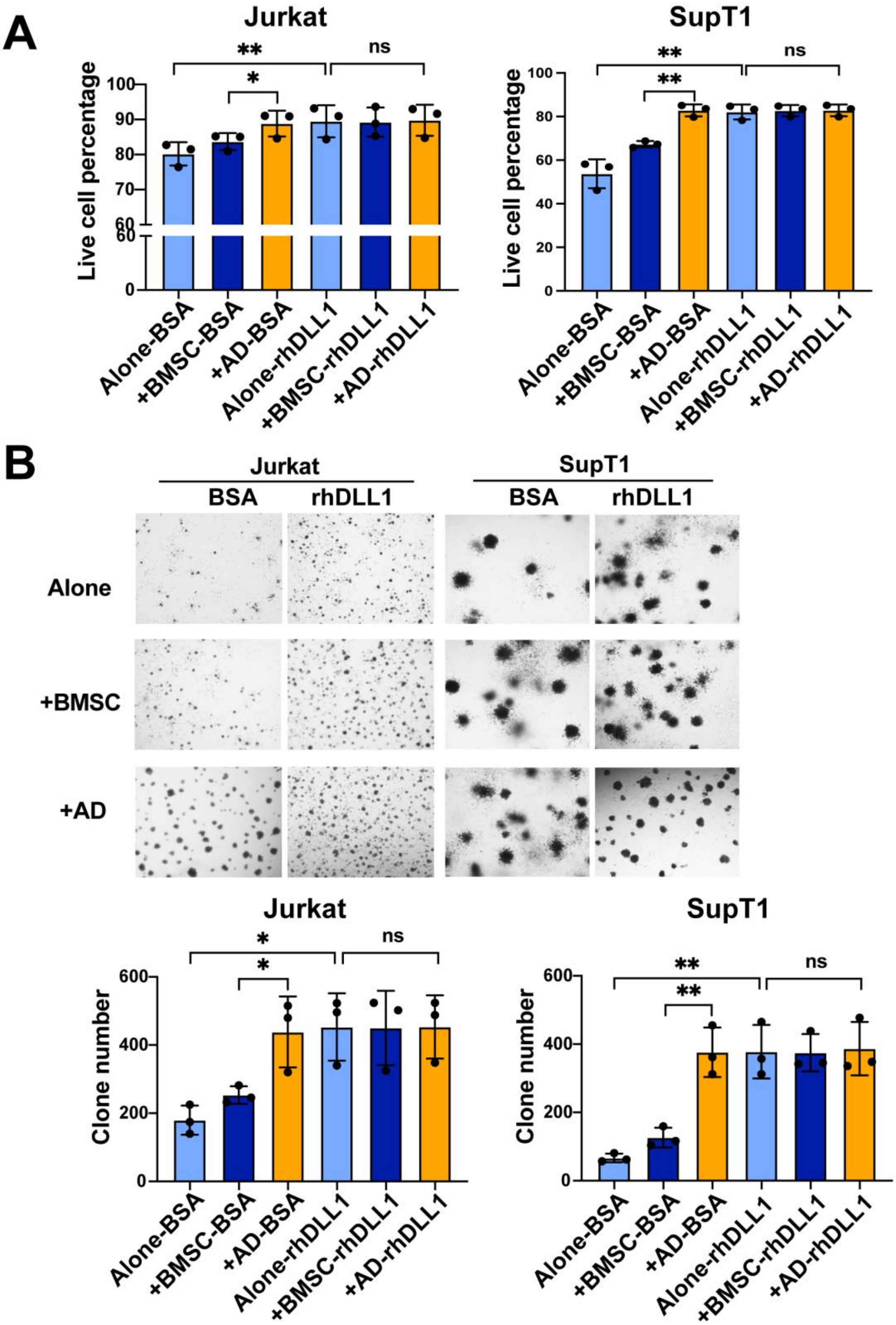

Supplementary Figure 2. BMSC-derived adipocytes support T-ALL cells via the binding of DLL1 and Notch1

(A) Representative cell apoptotic analysis of T-ALL cell lines from monoculture (alone), BMSC or AD cocultures in the presence of rhDLL1 and Ara-C. Each data point denotes a T-ALL patient BMSC sample. (B) The clone numbers of T-ALL cell lines from monoculture (alone), BMSC or AD cocultures under treatment with rhDLL1. Representative CFU micrographs are shown on the bottom. Each data point denotes a T-ALL patient BMSC sample.

Supplementary Table 1. Clinical information of T-ALL patients

	**Sex**	**Age (years)**	**WBC count (×10^9/L)**	**Hemoglobin (g/L)**	**Platelet count (×10^9/L)**	**Bone marrow blasts (%)**	**Induction chemotherapy**
**T-ALL01**	Male	39	7.99	59	83	84.92	COAP
**T-ALL02**	Female	30	8.7	130	144	40.25	COAP
**T-ALL03**	Male	32	440	115	156	92.48	VDLP
**T-ALL04**	Female	33	8.65	110	22	42.92	VDCLP
**T-ALL05**	Male	49	8.54	58	75	45.14	VDCLP
**T-ALL06**	Female	24	105.5	129	93	59.84	VDCLP
**T-ALL07**	Male	55	2.03	53	205	39.23	VDCLP
**T-ALL08**	Female	55	0.91	77	101	93.28	VDCLP
**T-ALL09**	Male	50	292	72	15	98.17	VDCLP
**T-ALL10**	Male	20	222	139	24	91.13	VICLP
**T-ALL11**	Male	54	3.4	107	218	70.45	VDCLP
**T-ALL12**	Male	23	320	138	180	95.8	VDCLP
**T-ALL13**	Male	45	72.08	71	25	95.82	VICLP
**T-ALL14**	Male	31	2.71	108	43	70.64	VDCLP
**T-ALL15**	Male	26	99.52	85	43	91.19	VDCLP
**T-ALL16**	Female	26	222.8	124	35	81.3	VDP
**T-ALL17**	Male	39	2.14	141	170	73.24	VDLP
**T-ALL18**	Male	31	246	128	220	94.12	VDCLP

**T-ALL19**	Male	31	20.65	81	37	81.37	VDCLP
**T-ALL20**	Male	16	86.06	105	30	85.88	VDCLP
**T-ALL21**	Male	46	61.88	72	9	30.94	VDCLP
**T-ALL22**	Male	50	69.37	72	89	91.51	VDCLP

Abbreviations: T-ALL, T-cell acute lymphoblastic leukemia; WBC, white blood cell; COAP, Cyclophosphamide+Vincristine+Cytarabine+Glucocorticoid; VDCLP, Vincristine+Daunorubicin+Cyclophosphamide+L-asparagine+Glucocorticoid; VICLP, Vincristine+Idarubicin+Cyclophosphamide+L-asparagine+Glucocorticoid; VDP, Vincristine+Daunorubicin+Glucocorticoid; VDLP, Vincristine+Daunorubicin+L-asparagine+Glucocorticoid
